# First record of the foraminiferal species *Ammonia confertitesta* in southeastern Baltic sea

**DOI:** 10.1038/s41598-025-18578-7

**Published:** 2025-09-29

**Authors:** Natalia Szymańska, Dhanushka Devendra, Ngoc-Loi Nguyen, Maria Holzmann, Agnieszka Garbień, Maria Stachowiak, Lech Kotwicki, Michael Lintner, Nina Keul, Katarzyna Melaniuk, Magdalena Łącka, Marek Zajączkowski, Jan Pawłowski

**Affiliations:** 1 Institute of Oceanology, Polish Academy of Sciences, Sopot, 81-712 Poland; 2 Department of Genetics & Evolution, University of Geneva, Geneva, 1205 Switzerland; 3 Faculty of Oceanography and Geography, University of Gdańsk, Gdynia, 81-378 Poland; 4 Department of Animal Taxonomy and Ecology, Adam Mickiewicz University, Poznań, 61-614 Poland; 5 Institute of Geological Sciences PAN, Kraków, 31-002 Poland; 6 Institute of Geosciences, Christian-Albrechts-Universität zu Kiel, Ludewig-Meyn-Str.10, 24118 Kiel, Germany

**Keywords:** Marine biology, Invasive species, Water microbiology

## Abstract

Early identification of non-indigenous species is important, as they have the potential to disrupt biogeochemical cycles, ecosystem stability, and trophic energy transfer in fragile ecosystems. Non-indigenous species may impact the functioning of the Baltic Sea ecosystem, the largest brackish water body in the world. This study presents the first record of the non-indigenous Asian foraminiferal species *Ammonia confertitesta* in the southeastern Baltic Sea. The species was recorded at 6 out of 14 sampling stations in the Gulf of Gdańsk, Poland, with one station sampled several times throughout the year, where *A. confertitesta* was consistently present. The species was also found in Puck Bay, a shallow western branch of the Gulf of Gdańsk. Our finding marks the first modern record of the genus *Ammonia* in the southeastern Baltic Sea. It provides new evidence for the rapid spread of *A. confertitesta* in European waters, highlighting its ability to thrive in environments where other calcareous foraminiferal species face difficulties. Morphometric comparisons with a population of the same species found in the western Baltic Sea shows that the Gulf of Gdańsk specimens are smaller. This may indicate that the species has reached its limits of environmental tolerance. However, given the increasing abundance of *A. confertitesta* in the Gulf of Gdańsk, it is important to monitor its further progression and investigate its long-term ecological impact on native species and the Baltic Sea ecosystem.

**Supplementary Information:**

The online version contains supplementary material available at 10.1038/s41598-025-18578-7.

## Introduction

Non-indigenous species (NIS) may influence the stability of the Baltic Sea ecosystem. This shallow coastal sea is strongly influenced by human activities due to inputs from several large European rivers, limited exchange with the open sea, and the resulting eutrophication and pollution. There are 229 non-indigenous and cryptogenic species reported in the Baltic Sea^1^. Most of these organisms are represented by groups such as annelids, arthropods, fish, and molluscs. There are also some phytoplankton species (mainly diatoms) listed among Baltic Sea NIS^1^. However, no data are available on benthic protist NIS in the Baltic Sea, although they have a high potential for colonizing new environments, due to the dominance of protists in ballast water transported biota^2^. Detecting the presence of benthic protist NIS is crucial, as these organisms play a key role in ecosystem functions such as nutrient cycling, energy transfer across trophic levels, and sediment stabilization. The currently changing Baltic Sea environment may create suitable niches for benthic protist NIS, which may influence local biodiversity and impact the composition and structure of benthic communities.

Among various protist groups, foraminifera are a major component of benthic communities. The ability of foraminiferal species to colonize new environments has been extensively studied^2,3^ and recognized as a potential to become NIS^4,5^. However, little is known about their capacity to spread to new regions. *Ammonia confertitesta* is characterized by a perforated calcareous test (shell) and belongs to one of the most common genera in coastal areas and shallow waters. First described from Chinese waters^6^ this species likely originated in Southeast Asia and has spread as NIS to various coastal areas around the world including the northwestern Atlantic^7^ the northeastern Pacific^8^ and Western Europe^9^.

The successful colonization of new regions by *A. confertitesta* is most likely due to its ability to adapt to a wide range of environmental conditions, particularly those influenced by human activity. Goetz, et al.^7^ have suggested that the species is transported via ballast water, a primary pathway for the dispersal of NIS. *Ammonia confertitesta* has shown tolerance to stressors such as low oxygen concentrations, low pH and heavy metal pollution, and is well adapted to brackish conditions^10,11^. Glock, et al.^12^ found that *A. confertitesta* could store large amounts of phosphate, which could provide an advantage in stressful environments and explain its opportunistic behaviour.

The first record of *A. confertitesta* in the Baltic Sea was from Kiel Bay^10^ followed by additional sightings north of Bornholm Island^9^. However, all these observations are from the western Baltic Sea, where the inflow of saline and oxygenated water from the North Sea supports relatively abundant and diverse foraminiferal assemblages. In contrast, foraminiferal abundance is much lower in the eastern and southeastern Baltic Sea, primarily due to its low salinity and limited inflow of North Sea waters. Foraminiferal records from this region are rare, with the only available data on species distribution coming from the work of Brodniewicz^13^ and Ponomarenko, et al.^14^. Given the ongoing environmental changes and the limited available data for the Baltic Sea—except for its western part—a current assessment of benthic foraminiferal assemblages is essential. In this study, we investigated the benthic foraminifera community in Gulf of Gdańsk and revealed an abundant population of *A. confertitesta*, which is the first record of this species in the southeastern Baltic Sea. Our observation is particularly noteworthy as it is based on investigation of shallow sediments (< 10 m) from Gulf of Gdańsk and Puck Bay - areas where calcareous foraminifera have not been previously recorded, and offers new insights into the distribution and cosmopolitan nature of *A. confertitesta*.

## Methods

### Sampling

Sediment samples were taken from fourteen stations in Gulf of Gdańsk and two stations in Kiel Bay. Stations 1–8 in Gulf of Gdańsk were sampled aboard the M/V *EDDA* using a Van Veen sediment grab on 09.07.2024, while stations 9–14 were sampled aboard S/Y *Oceania* using Van Veen sediment grab on 07.05.2024. Station 4 in Gulf of Gdańsk was additionally sampled from a pier using a handheld sediment grabber on 12.07.2023, 22.09.2023, 15.03.2024 and 06.06.2024. The Kiel Bay samples were taken using Van Veen sediment corer on 26.09.2024 aboard the ship R/V *Alkor*. Data on station coordinates is presented in Table S3 of the Supplementary Material. Either one or two days after collection, the upper two centimetres, circa 25 cm^3^ of sediment were analysed for foraminifera.

### Morphology assessment

At stations yielding a sufficient number of *A. confertitesta* (more than 25 specimens per sample), randomly selected individuals were analysed for test size, porosity, pore density and diameter following an established protocol by Petersen, et al.^15^ and using ImageJ software. Size is expressed in pixels and refers to the test area. For Scanning Electron Microscope (SEM) imaging, the tests were positioned with the spiral side facing upward and placed as horizontally as possible to minimize orientation bias. The SEM pictures were taken using a Hitachi TM4000Plus Tabletop Microscope. Both porosity and pore density were measured on the last chamber of the foraminiferal test on a 1280 × 960 pixel SEM image. Statistical analyses, including Kruskal-Wallis tests and ANOVA for test size, porosity, and pore density were performed using R version 4.1.1 with the *vegan* packages. Porosity is defined as percentage of an area covered by pores. Pore density is defined as number of pores per area (1280 × 960). Results of all morphometric analyses can be found in Tables S1 A-D in the Supplementary Material. Selected live specimens were dried on micropaleontological slides at ambient temperature and DNA barcoding analysis was performed at the University of Geneva.

### DNA extraction, PCR amplification and Sanger sequencing

Eight *Ammonia* specimens were photographed prior to extraction using a Leica M205C microscope fitted with a Leica DFC 450 C camera. Four individuals were sampled from Kiel Bay (isolates 22153–22156) and four individuals were collected from Gulf of Gdańsk (22157–22160). DNA was extracted individually using the Guanidine Buffer Solution^16^. Semi-nested polymerase chain reaction (PCR) protocol and thermal cycles were performed according to Holzmann^16^. The fragment represents the standard barcoding fragment in foraminifera^17^ and was amplified using primer pairs s14F3 (5’ACG CAM GTG TGA AAC TTG-3) - sB (5’TGA TCC TTC TGC AGG TTC ACC TAC-3) for the first amplification and s14F1 (5’AAG GGC ACC ACA AGA ACG C-3) – sB for the second amplification. Six PCR products were obtained and purified using the Roti Prep PCR Purification kit (Roth). Sequencing reactions were performed using the BigDye Terminator v3.1 Cycle Sequencing Kit (Applied Biosystems) and analysed on a 3130XL Genetic Analyzer (Applied Biosystems). The newly acquired sequences were deposited in the NCBI/GenBank database. Isolate and accession numbers are specified in Table S2 in the Supplementary Material.

### Phylogenetic analysis

The six obtained sequences were added to 20 *Ammonia* spp. sequences that are part of the curated ribosomal reference database of benthic foraminifera^16^. The alignment contains 26 sequences and 1102 sites were used for analysis. Sequences were aligned using the default parameters of the Muscle automatic alignment option as implemented in SeaView vs. 4.3.3^18^.

The Phylogenetic tree was constructed using maximum likelihood phylogeny (PhyML 3.0) as implemented in ATGC: PhyML^19^. An automatic model selection by SMS (Smart Model Selection)^20^ based on the Akaike Information Criterion (AIC) was used, resulting in a GTR + R (General Time Reversion + Gamma Distribution) substitution model being selected for the analysis. The initial tree is based on BioNJ. Bootstrap values (BV) are based on 100 replicates (Supplementary Material, Table S2).

## Results

### Foraminiferal occurrence

**Fig. 1:**
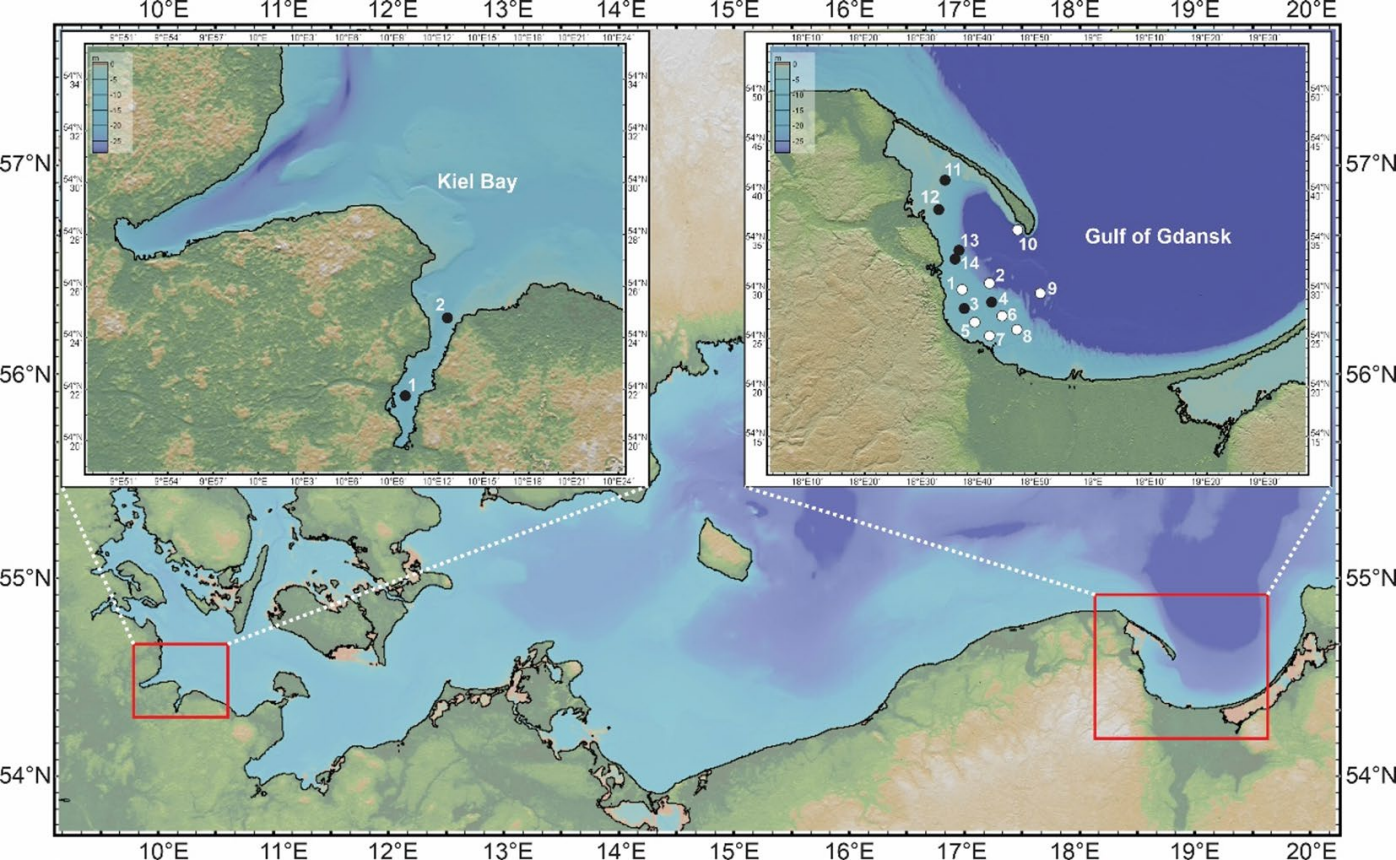
Map of study sampling sites (*Ammonia confertitesta* presence is indicated by black, filled dots). The map was generated using GeoMapApp software version 3.7.5 (http://www.geomapapp.org)^27^.

*Ammonia confertitesta* was found at 6 out of 14 sampling stations in Gulf of Gdańsk (Fig. 1). At stations 3 and 4, *A. confertitesta* was more abundant (91 and 76 specimens per sample in summer 2024, respectively) than at the other sites (Table 1), allowing for a comparative analysis with specimens from Kiel Bay. Specimens belonging to the agglutinated foraminiferal genera *Ammobaculites*, *Milliammina*, and *Reophax* were found at stations 11, 12, 13 and 14 in Gulf of Gdańsk.

**Table 1:** Foraminiferal abundance at Gulf of Gdańsk sampling stations (specimens per 25 cm^3^ of sediment). Stations where no specimen were found are excluded.

Station	Gulf of Gdańsk 3	Gulf of Gdańsk 4				Gulf of Gdańsk 11	Gulf of Gdańsk 12	Gulf of Gdańsk 13	Gulf of Gdańsk 14
Date of sampling	09.07.2024	12.07.2023	22.09.2023	06.06.2024	09.07.2024	07.05.2024	07.05.2024	07.05.2024	07.05.2024
No. of *Ammonia confertitesta* specimens	91	19	28	48	76	5	13	18	12
No. of *Ammobaculties* spp. *specimens*	0	0	0	0	3	0	0	4	1
No. of *Miliammina arenacea* specimens	0	0	0	2	0	0	0	0	0
No. of *Reophax sp.* specimens	0	0	0	0	2	0	0	0	0

### Morphometric analysis

**Fig. 2:**
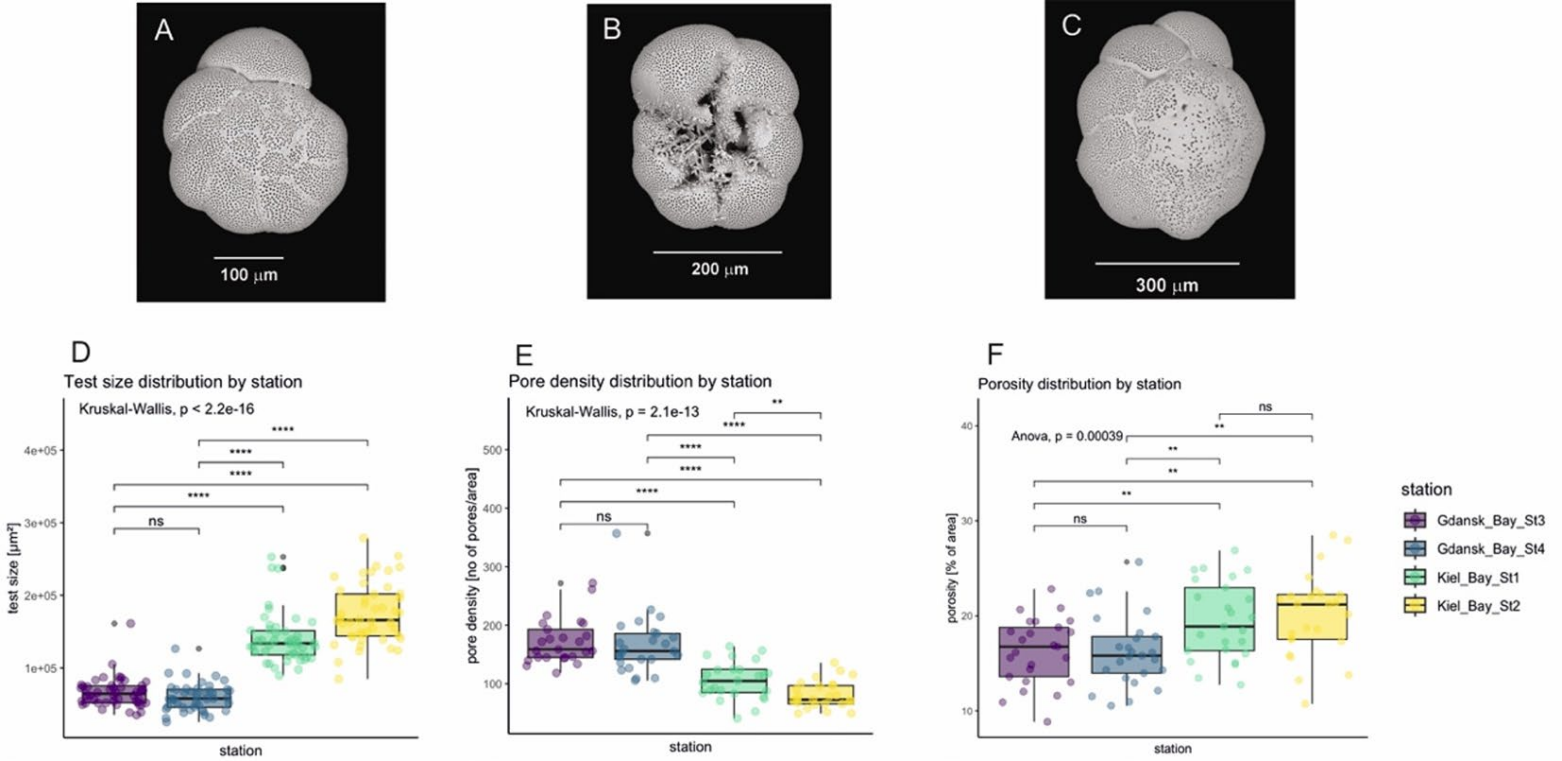
(**a**) *Ammonia confertitesta* from Gulf of Gdańsk, dorsal view. (**b**) *A. confertitesta* from Gulf of Gdańsk, ventral view. (**c**) *A. confertitesta* from Kiel Bay, dorsal view. (**d**) Test size distribution (µm²) of *A. confertitesta* at four sampling stations: stations 3 and 4 in Gulf of Gdańsk and stations 1 and 2 in Kiel Bay. (**e**) Porosity of *A. confertitesta* tests at four sampling stations, expressed as the percentage of area covered by pores in SEM images (3000 × magnification) of the final chamber. (**f**) Pore density of *A. confertitesta* tests at four sampling stations, expressed as the number of pores in a 1280 × 960 pixel area of an SEM image (3000 × magnification) of the final chamber. Significance for plots D, E, F is marked by * (* → *p* < 0.05; **→ *p* < 0.01; *** → *p* < 0.001; **** → *p* < 0.0001).

*Ammonia confertitesta* specimens from the Gulf of Gdańsk (Fig. 2A and B) are noticeably smaller than those from Kiel Bay (Fig. 2C), with an average size of approximately 63k µm^2^ and average diameter of circa 324 μm. In comparison, specimens from station 1 in Kiel Bay average 140k µm^2^ and diameter of 476 μm, while those from station 2 reach an average of 174k µm^2^ and diameter of 535 μm (Fig. 2D, Supplementary Material, Tables S1 A-D). No significant difference was found between stations 3 and 4 in the Gulf of Gdańsk, whereas significant differences were observed between all other station pairs (Kruskal-Wallis, *p* < 0.001, Fig. 2D).

Porosity measurements of the tests revealed that *A. confertitesta* specimens from Kiel Bay exhibited a higher pore coverage, averaging more than 20%, compared to approximately 17% in specimens from the Gulf of Gdańsk. We found statistically significant differences of pore coverage between stations in the Gulf of Gdańsk and Kiel Bay (ANOVA, *p* < 0.01), whereas we observed no difference in pore coverage in specimens within the same bay (*p* > 0.05, Fig. 2E). Specimens of *A. confertitesta* from Gulf of Gdańsk exhibited a larger number of pores than specimens from Kiel Bay. We observed a significant difference between sampling stations at Kiel Bay (Kruskal-Wallis *p* < 0.01), but not for those within the Gulf of Gdańsk (*p* > 0.05, Fig. 2F).

### Molecular data

The obtained sequences cluster within *A. confertitesta* that builds a strongly supported group (100% BV) branching as sister to a clade containing *A. corallinarum*,* A. neobeccarii*,* A. batava*,* A. pawlowskii* and *A. aberdoveyensis*. *Ammonia veneta* branches at the base of the tree. All species represented in the tree are strongly supported (99–100% BV).

## Discussion

The presence of *Ammonia confertitesta* in the Gulf of Gdańsk highlights its potential as a NIS and indicates its ongoing expansion in European waters. It confirms the spread of the species, previously observed at numerous sites in Atlantic coastal estuaries in France, where it has outcompeted native *Ammonia* species^21^. While it was previously found mainly in harbours, it is now also spreading along the English coast and has been reported in a large number of sites in the English Channel^9^. Records of this species in river estuaries and Kiel Bay in Germany^8–10^ indicate that it has reached the coastal waters of the Baltic Sea. Here we are the first to confirm its distribution is not limited to the western part of the Baltic Sea.

It has been suggested that *A. confertitesta* was introduced into European waters relatively recently. The earliest molecular record of *A. confertitesta* in the Wadden Sea dates from the late 1990s^17^. This was congruent with the first occurrence of *A. confertitesta* in Lake Grevelingen (The Netherlands), in the 1980s^22^. A study of sediment cores from the Loire Estuary is dating back the occurrence of this species to the 17th or 18th century^21^. However, this study was based on amorphometric analysis of *A. confertitesta*, which is challenging given its morphological similarity to other *Ammonia* species. The analysis of sediment ancient DNA from the cores would be necessary to confirm the early presence of this species in the Europe.

Prior to our study, there was no evidence of the presence of *A. confertitesta* in the modern southeastern Baltic Sea. In a comprehensive monograph of southern Baltic Sea foraminifera published in 1965, no *Ammonia* are reported from recent sediments^13^. The genus was present in this area in the fossil assemblage of the Littorina Sea (more than 4 000 years ago) at a site located on the northern Polish coastline, but not in Puck Bay or the shallow waters (< 10 m) of the Gulf of Gdańsk. Remarkably, no other calcareous foraminifera have been found in recent sediments of the Gulf of Gdańsk^13^ and sampling campaigns conducted prior to 2019 did not yield any calcareous foraminifera in the bay.

The absence of calcareous foraminifera in the Puck Bay and shallow waters of the Gulf of Gdańsk remain unclear. It may be linked to low salinity and consequently limited availability of carbonate or calcium ions. A recent gradual increase in calcium ions in the Baltic Sea, driven by anthropogenic input, could explain the successful colonization by calcifying non-indigenous species (NIS)^23^. This is evidenced by the rapid invasion of the region by the large, thick-shelled bivalve *Rangia cuneata*^24^. However, it is carbonate ions that have been identified as the limiting factor for calcification in the genus *Ammonia*^25^and the Vistula River supplies large amounts of carbonate ions into the Gulf of Gdańsk^26^.

Other environmental factors, such as water quality or food availability may also enhance the dispersion of *A. confertitesta* in the Gulf of Gdańsk. The recent southeastern Baltic Sea is rich in diatoms and its sediments are characterized by high loads of organic matter, the primary food sources for foraminifera^14^. Indeed, SEM images of *A. confertitesta* from the Gulf of Gdańsk reveal diatom frustules near the aperture, which could indicate that diatoms serve as a food source for this species (Fig. 2B). Alternative explanations for the appearance of *A. confertitesta* include the recent improvement in environmental quality in the region, resulting from the implementation of various policies regulating sewage treatment and the use of artificial fertilizers, or the broader consequences of modern climate change.

Our study confirms that *A. confertitesta* exhibits exceptional adaptability to environmental conditions^11^. The fact that the specimens from Gulf of Gdańsk are smaller than those from Kiel Bay, suggests that the species may be approaching - or may have already reached its environmental tolerance limits in the Gulf of Gdańsk. At the same time, specimens from the Gulf of Gdańsk exhibit lower porosity and higher pore density than those from Kiel Bay, suggesting that they experience less stress related to low oxygen conditions^15^. Higher porosity facilitates better gas exchange in low-oxygen environments, while lower pore density may result from fewer pores occupying the same surface area.

If the species is indeed at its environmental tolerance limit, it may be more vulnerable to future environmental changes. Consequently, its abundance could decline or the species might disappear, particularly if climate change continues to adversely affect the physicochemical properties of local water masses (e.g. increased temperatures, decreased oxygen levels, or increased acidity in bottom waters). Although there are currently no studies on the negative environmental impacts of NIS foraminifera on ecosystems, monitoring the abundance of *A. confertitesta* and tracking its spread remains essential to assess its effects on native communities.

Given the historical low abundance of calcareous foraminifera in the southeastern Baltic Sea, the presence of *A. confertitesta* may represent a recent, yet significant shift in the ecosystem of the region. Further studies are also needed to understand the long-term ecological impacts of *A. confertitesta* on native species and ecosystem stability in the Baltic Sea.

## Supplementary Information

Below is the link to the electronic supplementary material.

Supplementary Material 1

Supplementary Material 2

Supplementary Material 3

## Data Availability

Data generated and analysed during this study are included in this published article as Supplementary Material (Tables S1A–D, S2, and S3). Additional SEM photographs are available from the corresponding author upon reasonable request.
